# Spleen Abscess as Malaria Complication

**DOI:** 10.3201/eid1203.050311

**Published:** 2006-03

**Authors:** Sandro Contini, Harold R.N. Lewis

**Affiliations:** * University of Parma, Parma, Italy;; † "Emergency" Hospital Goderich, Freetown, Sierra Leone

**Keywords:** spleen abscess, malaria, Plasmodium falciparum, letter

**To the Editor:** Changes in spleen structure, frequently encountered during malaria, may result either in a simple asymptomatic enlargement or in serious complications such as hematoma, rupture, or infarction (*1**–**3*). Hematoma or infarction of the spleen might be followed by the development of a splenic abscess, a clinical condition that has been reported in only 1 patient, to our knowledge (*4*).

A 21-year-old woman sought treatment at the hospital outpatient department of "Emergency," an Italian nongovernmental organization (NGO) in Freetown, Sierra Leone in May 2004, reporting malaise and persisting dull abdominal pain, accompanied by isolated episodes of spiking fever. Several recurrent malaria attacks (*Plasmodium falciparum*) had been reported by this patient in the last 2 months. At physical examination, conjunctival pallor and a tender, enormously distended abdomen were observed. A large abdominal mass, extending from the xiphoid process to the pubis, was palpable. Lymph nodes (neck, axillary, inguinal) were normal. Laboratory features showed severe anemia (hemoglobin 62 g/L; hematocrit 0.24), with low platelet count (90 × 10^3^/μL) and elevated leukocyte count (130 × 10^3^/μL), together with a moderate increase in liver enzymes (both aspartate aminotransferase and alanine aminotransferase were more than twice the upper limit of normal values). No malaria parasites were observed on blood smear at admission. Results of an HIV test were negative as were results of a sickle cell test, and hemoglobin electrophoresis results were normal. Other evident septic foci (e.g., typhoid fever, urinary tract infection, osteomyelitis) were excluded. Stool and urine examination excluded schistosomiasis. Blood cultures were not available.

An abdominal radiograph showed intraperitoneal fluid without distension of the bowel, whereas results of an abdominal ultrasound, performed in a private laboratory, diagnosed a large tumor on the left ovary. After receiving a blood transfusion (2 units) and intravenous antimicrobial drug treatment (ampicillin 500 mg 4 times/day, chloramphenicol 1 g 2 times/day, and metronidazole 500 mg, 2 times/day), the patient was scheduled for an exploratory laparotomy. Abdominal paracentesis was performed the day before surgery, and 2 L of thick brownish fluid was extracted.

An explorative laparotomy found ≈3 L of infected fluid in the peritoneal cavity. Widespread fibrin membranes covered thickened ileal loops. The mass was found to consist almost entirely a very large abscess on the spleen (Figure), which contained ≈5 L of pus. Dense adhesions were observed between the spleen, greater omentum, liver, and ileal loops. The liver was normal, and portal hypertension was not found. After splenectomy, the spleen's length was found to be 48 cm, and its weight was 6 kg. On histologic examination, splenic tissue was found to have been replaced by congested inflammatory infiltrates and fibrotic tissue. Leishmaniasis was excluded at microscopic examination. The patient completely recovered after surgery.

**Figure:**
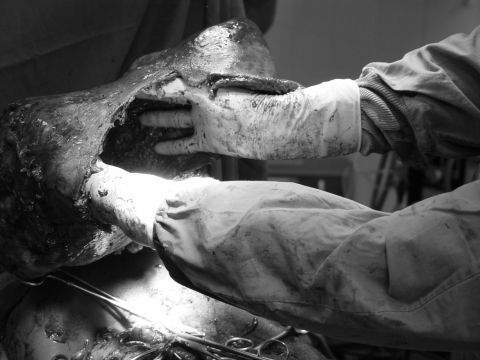
Dimensions of the abscess cavity are shown during the operation.

An enlarged spleen is found in 50% to 80% of malaria patients (*1*), while only 25 cases of splenic rupture have been reported since 1960 (0%–2% in natural occurring infection) (*5*). A break of a contained hematoma is usually involved in splenic rupture, which occurs almost exclusively during acute infection and the primary attack (*6*). The incidence of splenic hematoma without rupture is unknown (*2*).

Spleen infarction is rarer than rupture and may go unnoticed. Only 9 documented cases of splenic infarction associated with malaria have been reported (*3*), all consequent to *P*. *falciparum* infection (except in 1 patient who was coinfected with *P*. *vivax* and 2 cases in which the etiologic agent was unknown). Splenic rupture following infarction has not yet been described.

Recently, an abscess of the spleen caused by *Salmonella enterica* serovar Enteritidis has been reported as a complication of *P*. *falciparum* malaria (*4*) and, to our knowledge, is the only case in the literature definitely related to *Plasmodium* infection. Indeed, splenic hematoma or infarction, together with the humoral and cellular immunodepression due to malaria, might well be predisposing factors for bacterial (e.g., salmonellae) colonization of the spleen from the gut, as likely happened in this patient, although cultures of the pus, blood, or intraabdominal fluid were not performed. Bacteremia caused by nontyphoidal salmonellae was significantly associated with malaria parasitemia (*7*), and splenic abscess has been recently reported as an atypical presentation of salmonellosis (*8*). Splenic abscesses caused by *Salmonella* infection usually occur on preexisting lesions (*4*) and have been increasingly reported recently (*9*).

Because of its insidious symptoms, a spleen abscess remains a diagnostic challenge in developing countries, where ultrasounds and computed tomographic scans are not easily accessible. Moreover, as in our patient, a spleen abscess is unlikely to develop as an immediate complication of malaria.

While splenectomy was the only possible treatment in this patient, a conservative approach, whenever possible, is always desirable, especially in the tropics, where the exposure to infective agents is particularly widespread. The overall prognosis of splenic abscesses remains discouraging, with 13%–16% of cases resulting in death (*9*), mainly consequent to late diagnosis and admission to a hospital. The growing volume of international travel will likely lead to an increase in the incidence of splenic complications in malaria patients, even in areas where the disease is not endemic. Therefore, clinicians should always keep the possibility of a superimposed abscess in mind.
